# The Double-Faceted Role of Leucine-Rich Repeat Kinase 2 in the Immunopathogenesis of Parkinson’s Disease

**DOI:** 10.3389/fnagi.2022.909303

**Published:** 2022-05-11

**Authors:** Mengfei Zhang, Chaoyi Li, Jie Ren, Huakun Wang, Fang Yi, Junjiao Wu, Yu Tang

**Affiliations:** ^1^Department of Geriatrics, Xiangya Hospital, Central South University, Changsha, China; ^2^Aging Research Center, Xiangya Hospital, Central South University, Changsha, China; ^3^National Clinical Research Center for Geriatric Disorders, Xiangya Hospital, Central South University, Changsha, China; ^4^Department of Rheumatology and Immunology, Xiangya Hospital, Central South University, Changsha, China; ^5^Provincial Clinical Research Center for Rheumatic and Immunologic Diseases, Xiangya Hospital, Central South University, Changsha, China; ^6^Department of Neurology, Xiangya Hospital, Central South University, Changsha, China; ^7^The Biobank of Xiangya Hospital, Central South University, Changsha, China

**Keywords:** Parkinson’s disease, LRRK2, neuroinflammation, microglia, immune function

## Abstract

*Leucine-rich repeat kinase 2* (*LRRK2*) is one of the most common causative genes in Parkinson’s disease (PD). The complex structure of this multiple domains’ protein determines its versatile functions in multiple physiological processes, including migration, autophagy, phagocytosis, and mitochondrial function, among others. Mounting studies have also demonstrated the role of LRRK2 in mediating neuroinflammation, the prominent hallmark of PD, and intricate functions in immune cells, such as microglia, macrophages, and astrocytes. Of those, microglia were extensively studied in PD, which serves as the resident immune cell of the central nervous system that is rapidly activated upon neuronal injury and pathogenic insult. Moreover, the activation and function of immune cells can be achieved by modulating their intracellular metabolic profiles, in which LRRK2 plays an emerging role. Here, we provide an updated review focusing on the double-faceted role of LRRK2 in regulating various cellular physiology and immune functions especially in microglia. Moreover, we will summarize the latest discovery of the three-dimensional structure of LRRK2, as well as the function and dysfunction of LRRK2 in immune cell-related pathways.

## Introduction

**FIGURE 1:**
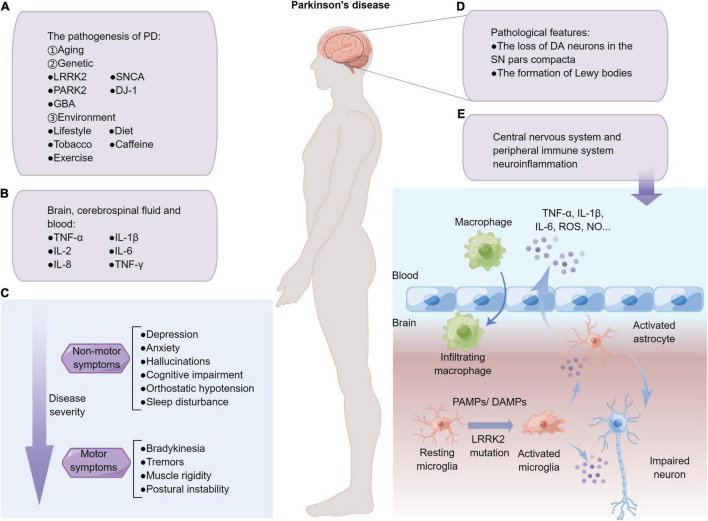
Overview of PD and neuroinflammation. **(A)** The pathogenesis factors of PD mainly include aging, genetic and environmental factors. **(B)** PD is a disease of systemic inflammation with pro-inflammatory factors that have been detected in the human brain slices, cerebrospinal, and blood. **(C)** Patients with PD often present with non-motor symptoms, which often precedes the development of motor symptoms. **(D)** Two major pathological features of Parkinson’s patients. **(E)** PAMPs/DAMPs stimulation, as well as PD-related gene mutation such as *LRRK2*^*G*2019*S*^, promote resting microglia skewing into pro-inflammatory phenotypes, which secrete excessive pro-inflammatory factors that may damage neurons and activate astrocytes in the milieu. The damaged blood-brain barrier of PD patients further causes neuroinflammation to diffuse in the CNS and peripheral system, and develop from acute inflammation to systemic chronic inflammation.

Parkinson’s disease (PD) is the second most common neurodegenerative disease after Alzheimer’s disease. PD affects at least 1% of people over the age of 65 and at least 4% of people over the age of 80 (Elbaz et al., 2016). The major pathological features of PD patients are the loss of dopaminergic (DA) neurons in the substantia nigra (SN) pars compacta, as well as the abnormal accumulation of α-synuclein that leads to the formation of Lewy bodies (Figure 1). Furthermore, PD is a progressive degenerative disease, a classic clinical motor syndrome characterized by bradykinesia, tremors, muscle rigidity, and postural instability. In addition to typical motor symptoms, PD patients also manifested several non-motor symptoms such as depression, anxiety, hallucinations, cognitive impairment, orthostatic hypotension, or sleep disturbance (Hussein et al., 2021; Figure 1). Clearly, the clinical diagnosis of PD is mainly based on the patient’s clinical symptoms, medical history, and responses to dopamine drug treatment. Of the approximately 145 PD treatments currently in clinical trials, 39% of the trials focus on long-term disease-modifying treatments, and the rest focus on short-term symptom relief treatments (McFarthing et al., 2020). What’s more, numerous clinical and basic research have linked autoimmune diseases, impaired cellular and humoral immune responses, inflammatory cell activation, and immune dysregulation to the pathogenesis of PD (Tan et al., 2020). Although the pathogenesis of PD has not been fully elucidated, there is increasing evidence indicating that PD is an immune-mediated inflammatory disorder.

The pathogenesis of PD is yet to be fully elucidated. Apart from old age, other factors such as environmental factors and genetic deficiencies also contribute to the degeneration of DA neurons (Ascherio and Schwarzschild, 2016; Figure 1). Although the majority of PD patients have sporadic forms, with only 5–10% hereditary (Rocha et al., 2018), genome-wide association studies (GWAS) have reported a critical role for genetic variants that might contribute to idiopathic PD pathogenesis (Nalls et al., 2014). The pathology of PD not only exhibited on DA neurons, but also on non-neural cells, such as glia cells. Specifically, microglia-mediated neuroinflammation is a prominent hallmark of PD. Interestingly, most of the PD pathogenic genes identified so far, such as α*-Synuclein*, *Parkin*, *DJ-1*, *LRRK2*, and *GBA*, are expressed in microglia cells, albeit with complicated and even controversial effects (Martin et al., 2011; Dzamko et al., 2015). Studying the functions of PD-related genes, such as *LRRK2*, is of great significance for delving the onset and development of neurodegenerative diseases (Dachsel and Farrer, 2010). Currently, delivering LRRK2 kinase inhibitors such as DNL151 has become an important avenue for treating LRRK2 diseased patients (Ding and Ren, 2020). Mutations in *LRRK2* are a relatively common cause of familial late-onset PD, and also linked to the more numerous sporadic PD, suggesting that understanding LRRK2-associated mechanisms might be a gateway to exploring sporadic PD (Rocha et al., 2022). Notably, the multi-domain structure of LRRK2 renders its versatile functions in multiple physiological processes, including migration, autophagy, phagocytosis, and mitochondrial function, among others. In this review, we therefore summarized the latest progress in the double-faceted role of LRRK2 in regulating various cellular physiology and particularly immune functions.

## Microglial Activation and Systemic Inflammation

Microglia, as resident immune cells in the central nervous system (CNS), generally interact with other cells in the milieu and play a crucial role in maintaining brain homeostasis. In the normal brain, microglia are considered “resting” that constantly surveil the brain microenvironment and actively contact neuron synapses (Kettenmann et al., 2013). However, microglia could be activated by any type of pathological event or changes in brain homeostasis, including various PAMPs and DAMPs, and rapidly proliferate and accumulate at the injury site, where they engulf dead cells and secrete pro-inflammatory factors such as TNF-α, IL-1β, IL-6, as well as reactive oxygen species (ROS) and nitric oxide (NO) (Sanchez-Guajardo et al., 2013; Wolf et al., 2017). However, the persistent over-activated phenotypes of microglia are detrimental to resident neurons, so that the activation and subsequent resolution of microglia-mediated neuroinflammation are strictly controlled (Perry and Teeling, 2013; Marinelli et al., 2019). Specifically, under the diseased context, chronic pathological factors such as repeated exposure to environmental toxins/PAMPs/DAMPs, genetic susceptibility, as well as abnormal immune responses, might exist. Meanwhile, increased levels of pro-inflammatory cytokines were observed in the brain, cerebrospinal fluid, and blood of PD patients, especially tumor necrosis factor-α (TNF-α), interleukin-1β (IL-1β), IL-2, IL-6, IL-8, and interferon-γ (IFN-γ) (Dzamko et al., 2015; Marogianni et al., 2020; Figure 1). As such, the over-activation of microglia may be prolonged, which further triggers a vicious cycle of chronic neural degeneration and pro-inflammation (Newcombe et al., 2018). Furthermore, microglia have been identified as key regulators of synaptic remodeling and brain wiring. Studies have shown that GABA initiates a transcriptional synapse remodeling program within GABA-receptive microglia, promoting direct network connections between neurons and microglia (Favuzzi et al., 2021). Recent studies have shown that microglia are involved in regulating synaptic remodeling and pruning through different molecular mechanisms, such as the cytokine interleukin-33 (IL-33) (Nguyen et al., 2020), phosphatidylserine (Scott-Hewitt et al., 2020), and complement component 3 (C3) (Werneburg et al., 2020). Moreover, Xia et al. (2021) found that the interaction of α-synuclein and toll-like receptor 2 (TLR2) of microglia leads to the phagocytosis of α-synuclein and further activates microglia. During neuroinflammation and neurodegeneration, complex mechanisms of crosstalk between astrocytes and other cells also occurred in the CNS (Linnerbauer et al., 2020). Studies have shown that interaction of astrocytes and microglia favors the clearance of amyloid-β and α-synuclein aggregates, highlighting the important role of glial crosstalk in the progression of PD (Rostami et al., 2021). Therefore, targeting microglia-mediated neuroinflammation might produce promising therapeutic benefits (Prinz et al., 2019).

Notably, neuroinflammation of the CNS and peripheral immune system are closely associated that act in synergy to the pathogenesis and development of PD (Tansey and Goldberg, 2010; Gelders et al., 2018; Figure 1). As one of the most sophisticated organs, the brain exists extensive bi-directional communication between resident immune cells with the peripheral immune system, the disruption of which could lead to cognitive and behavioral disorders (Filiano et al., 2015; Louveau et al., 2015; Kipnis, 2016). Systemic chronic inflammation is the pathological basis of neurodegenerative diseases including PD (Furman et al., 2019). When the risk factors are eliminated but the inflammatory response continues, it will lead to the occurrence of immune tolerance (Straub, 2017). It is known that macrophages and microglia play an important role in communication between the peripheral immune system and the CNS. Importantly, PD-related genes including *LRRK2* have been confirmed to exist both in CNS immune cells and peripheral immune cells; the communication between the two immune systems carrying PD mutations might render the brain easily reaching the critical threshold of inflammation, thereby exacerbating disease development (Huang et al., 2021).

## Leucine-Rich Repeat Kinase 2: Structure and Role in the Immune Cells

### The Structure and Function of Leucine-Rich Repeat Kinase 2

**FIGURE 2:**
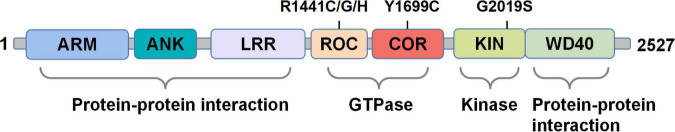
The domain structure of the LRRK2 protein. The domains are divided into protein-protein interaction, GTPase, kinase, and protein-protein interaction according to their functions. PD-associated mutations mentioned in the review are depicted on top.

Although the underlying mechanisms of pathogenic mutations contribute to PD are yet fully investigated, the involvement of *LRRK2* in the pathogenesis of PD has been continuously established. LRRK2 is expressed at a comparatively low level in the brain, but is highly expressed in the lung, spleen, kidney, and peripheral immune cells such as neutrophils, monocytes, and dendritic cells (Biskup et al., 2007; Dzamko, 2017; Lee et al., 2017). Di Maio et al. (2018) have shown that, independent of mutations, wild-type (WT) *LRRK2* plays a role in idiopathic PD and that LRRK2 kinase inhibitors can be used to treat idiopathic patients without *LRRK2* mutations. *LRRK2* mutations are the most frequent cause of autosomal dominant PD, accounting for approximately 3% of all cases (Paisan-Ruiz et al., 2004; Zimprich et al., 2004). The LRRK2 protein is a complex multi-domain protein with a size of 286 kDa, which contains seven domains, including armadillo repeat motif (ARM), ankyrin repeat (ANK), leucine-rich repeat (LRR), ras-of-complex (ROC), C-terminal of ROC (COR), kinase (KIN), and WD40 domains (Mills et al., 2018; Figure 2). The nature of large size and complex domains of LRRK2 protein thus might produce both great challenges and immense significance for medical research (Mata et al., 2006; Rui et al., 2018). Moreover, the structural differences between human and mouse LRRK2 determine the diverse characteristics of the LRRK2 protein. The mouse model is widely used as the major PD animal model for studying LRRK2 functions, which remains different from the actual clinical situation (Langston et al., 2019). Newly identified structures of LRRK2, as well as a better understanding of the interaction between LRRK2 and microtubules, canthus help elucidate the role of LRRK2 in the pathogenic mechanism of PD (Leschziner and Reck-Peterson, 2021).

Numerous studies have revealed that *LRRK2* mutants related to the pathogenesis of PD could lead to the over-activation of LRRK2 protein, in which G2019S and R1441C/G are the most common ones (Li et al., 2014). Specifically, the G2019S mutation is located at the kinase domain, while the R1441C/G mutations are located at the ROC GTPase domain (Iannotta et al., 2020). Functional alterations associated with pathogenic *LRRK2* mutations include, but are not limited to, alterations in vesicular trafficking and cytoskeleton dynamics, autophagy and lysosomal degradation, neurotransmission, mitochondrial function, and immune/microglial responses (Herzig et al., 2011; Chan and Tan, 2017; Cookson, 2017; West, 2017; Chen et al., 2018a; Shihabuddin et al., 2018; Cresto et al., 2019). The elaborated analysis of the direct relationship between the structure and function of LRRK2 would thus be helpful in the treatment of PD (Zhang et al., 2019; Deniston et al., 2020; Bolz et al., 2021). A recent study demonstrated that pathological α-synuclein activates LRRK2 expression and kinase activity in monocytes, inhibition of which may attenuate the pro-inflammatory monocyte responses in the brain (Xu et al., 2022). It is worth noting that the treatment of PD is not limited to modulating the LRRK2 kinase activity, other physiological processes mediated by LRRK2 such as autophagy could also be potential therapeutic targets (Wojewska and Kortholt, 2021). To determine how LRRK2 protein levels regulate inflammatory cytokine/chemokine levels in human immune cells, Ahmadi Rastegar et al. (2022) demonstrated that the *LRRK2*^*G*2019*S*^ mutation may aggravate inflammation following TLR activation in the differentiated monocytes and macrophages from human induced pluripotent stem cells (iPSCs), whereas LRRK2 kinase inhibitors manifest limited effect on TLR-mediated inflammation. More importantly, unlike other PD-related genes, hereditary and sporadic PD carrying *LRRK2* mutations have a high degree of consistency in clinical characteristics and treatment response (Kett and Dauer, 2012; Tolosa et al., 2020). Based on these, the *LRRK2* gene has become an important target for studying both hereditary and sporadic PD.

The G2019S mutation in the KIN domain of LRRK2 leads to increased kinase activity plausibly explaining the abnormal LRRK2 kinase activity. However, current studies still have many puzzles in explaining how mutations in the ROC and COR domains alter the function of the LRRK2 protein. Therefore, there is an urgent need for high-resolution LRRK2 structures to facilitate a deep understanding of the functions of individual domains. In the past few years, the high-resolution structures of LRRK2 protein have been successfully revealed, by using a combination of *in vitro* and *in situ* techniques (Deniston et al., 2020; Watanabe et al., 2020; Myasnikov et al., 2021; Tokars et al., 2021; Ullrich, 2021). Specifically, Weng et al. (2022) generated a comprehensive dynamic allosteric map of the C-terminal domain of LRRK2 (LRRK2RCKW) using hydrogen-deuterium exchange mass spectrometry (HDX-MS) and molecular dynamics (MD) simulations, while confirming that the kinase domain is a central hub for conformational control. Another study has obtained the structure of the mutant *LRRK2*^*I*2020*T*^ at the *in situ* level of the cell, which forms a double helix structure around microtubules (Watanabe et al., 2020). This study later constructed a microtubule atom model related to LRRK2, confirming that the closed conformation of the LRRK2 N-terminal catalytic structure can block the movement of the microtubule motor, whereas kinase inhibitors can interfere with the obstructive effect of LRRK2 (Deniston et al., 2020).

It is well-established that LRRK2 has three quaternary structures: monomer, dimer, and oligomer, which were found to display different biological activities. Tokars et al. (2021) cracked the structure of LRRK2 monomer, dimer and mutant G2019S based on the high-resolution structure of the full-length human LRRK2, and thus explained the internal structure, assembly and activity regulation mechanism of LRRK2. Myasnikov et al. (2021) further confirmed that COR-mediated single point mutations at the LRRK2 dimer interface can eliminate the pathogenic filaments formed by LRRK2. Specifically, the link between LRRK2 and microtubules have shed new insights into exploring the exact functions of LRRK2 and its inhibitors.

### Leucine-Rich Repeat Kinase 2 Expression in Immune Cells

**FIGURE 3:**
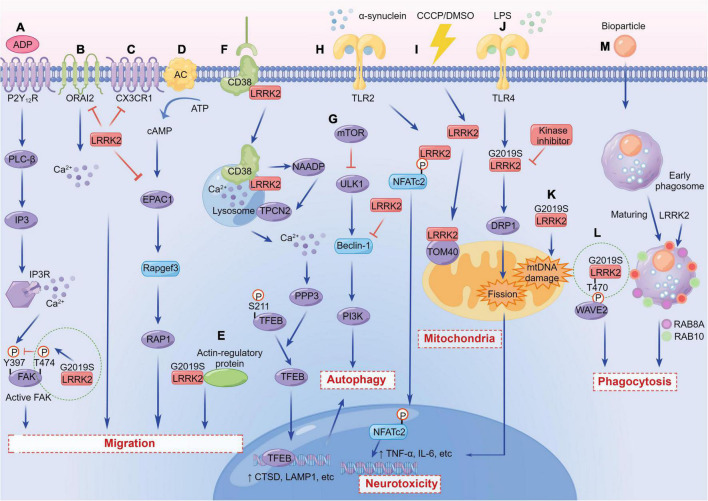
The versatile functions of LRRK2 in immune cells. **(A)** *LRRK2*^*G*2019*S*^ regulates microglial motility through inhibition of FAK. **(B)** LRRK2 suppresses the migration of microglia and enhances microglial inflammation by inhibiting the activity of CX3CR1. **(C)** LRRK2 modulates dendritic cells migration by interfering with ORAI2. **(D)** LRRK2 suppresses EPAC-1 activity, further restricting motility in macrophages. **(E)** *LRRK2*^*G*2019*S*^ enhances chemotaxis through enhanced interaction with actin-regulatory proteins in myeloid cells. **(F)** LRRK2 regulates autophagy by mediating the physiological CD38-LRRK2-TFEB signaling axis in B-lymphocytes and macrophages. **(G)** LRRK2 inhibits Beclin-1-induced macroautophagy independently of mammalian target of rapamycin (mTOR) and Unc-51-like kinase 1 (ULK1). **(H)** LRRK2 promotes a neuroinflammatory cascade by selectively phosphorylating and inducing nuclear translocation of the NFATc2. **(I)** WT and *LRRK2*^*G*2019*S*^ associate and co-localize with subunits of the TOM complex, either under DMSO or carbonyl cyanide m-chlorophenyl hydrazine (CCCP) conditions. **(J)** LRRK2 promotes microglial mitochondrial alteration *via* DRP1 in a kinase-dependent manner, initiating pro-inflammatory responses. **(K)** *LRRK2*^*G*2019*S*^ causes a significant increase in mtDNA damage in PD patient-derived immune cells. **(L)** *LRRK2*^*G*2019*S*^ binds and phosphorylates WAVE2 at Thr470, stabilizes and prevents its proteasomal degradation, and increases WAVE2-mediated phagocytosis. **(M)** LRRK2 is required for RAB8A and RAB10 recruitment to phagosomes in macrophages.

To understand the physiological and pathological functions of LRRK2, it is important to recognize the role of LRRK2 in different types of immune cells (Table 1). Most previous efforts to elucidate the LRRK2 functions during neuroinflammation have been focused on microglia, which is the first barrier of the brain’s innate immune system. A recent study revealed that LRRK2 mediated the neurotoxicity of microglia through phosphorylation, interaction and activation of the nuclear factor of NFATc2 in the mouse model of synucleinopathies (Kim et al., 2020; Figure 3). LRRK2-NFATc2 signaling axis may be a new therapeutic target for PD. Advances in the study of LRRK2 as a target for the treatment of PD bring hope to patients who are deeply troubled and harmed by the disease. Various studies have shown that LRRK2 mRNA and protein expression levels in human and rodent microglia were upregulated upon stimulation with lipopolysaccharide (LPS) *in vitro* (Miklossy et al., 2006; Gillardon et al., 2012; Moehle et al., 2012; Russo et al., 2015, 2019). Similarly, the LRRK2 expression in both human pluripotent stem cell (hPSC)-derived macrophages and microglia was enhanced in a time-dependent manner after IFN-γ treatment (Lee et al., 2020; Panagiotakopoulou et al., 2020). However, there also exhibits conflicting results. Several studies failed to determine the expression of LRRK2 in microglia from WT mice (Biskup et al., 2006; Higashi et al., 2007b; Westerlund et al., 2008), neither in brain slices of PD patients and healthy controls (Higashi et al., 2007a; Hakimi et al., 2011; Sharma et al., 2011; Dzamko et al., 2012, 2017). In another study, microglia isolated from rodent brains cannot induce the LRRK2 expression after LPS stimulation (Kozina et al., 2018).

**TABLE 1:** The LRRK2 expression in immune cells.

Species	Cell types	Treatment	LRRK2 expression	References
Human	Astrocytes, oligodendroglia, microglia, neuroblastoma cell	N/A	RNA levels detected	Miklossy et al., 2006
Mouse	Primary microglia (*LRRK2*^*R*1441*G*^)	LPS/IFN-γ	↑LRRK2 protein	Gillardon et al., 2012
Mouse, rat	Microglia, primary microglia	N/A	↑LRRK2 activity and expression	Moehle et al., 2012
Mouse	Primary microglia	LPS	↑LRRK2 mRNA	Russo et al., 2019
Mouse	BV2	LPS	No change	Russo et al., 2015
Human	hPSC-derived macrophages and microglia	IFN-γ	↑LRRK2 protein	Lee et al., 2020
Human	iPSC-derived microglia (*LRRK2*^*G*2019*S*^)	IFN-γ	↑LRRK2 mRNA and protein levels	Panagiotakopoulou et al., 2020
Rat	Microglia	N/A	Not detected	Biskup et al., 2006
Human	Microglia	N/A	Not detected	Higashi et al., 2007b
Rat	Microglia	N/A	Not detected	Westerlund et al., 2008
Human	Astrocytes, microglia, oligodendrocytes	N/A	Not detected	Sharma et al., 2011
Mouse	BMDMs, RAW264.7	LPS	↑Phosphorylation at Ser910 and Ser935, no change in total protein	Dzamko et al., 2012
Human	The postmortem brain tissue of PD patients	N/A	No change	Dzamko et al., 2017
Mouse	Microglia (*LRRK2*^*R*1441*G*^)	N/A	Not detected	Kozina et al., 2018
Human	Macrophages, B-lymphocytes, and CD103-positive dendritic cells (patients with CD and ulcerative colitis undergoing colonoscopy)	IFN-γ	↑LRRK2 mRNA and protein levels	Gardet et al., 2010
Human	Peripheral blood mononuclear cells	IFN-γ	↑LRRK2 mRNA and protein levels	Thevenet and Pescini Gobert, 2011
Human	B cells, T cells, CD16^+^ monocytes (PD patients)	N/A	↑LRRK2 protein	Cook et al., 2017
Human	Primary neutrophils, monocytes, γδ-type T cells, B cells, NK cells, CD4^+^ T cells, and CD8^+^ T cells (healthy donors)	N/A	LRRK2 mRNA: neutrophils > monocytes > B cells	Shutinoski et al., 2019
Human	THP-1 cells were differentiated into macrophage-like cells	IFN-γ	↑LRRK2 mRNA and protein	Kuss et al., 2014
Human	Monocyte subpopulations, in lymphoid B-cells (PD patients)	N/A	↑LRRK2 protein	Bliederhaeuser et al., 2016
Human	Neutrophils (PD patients)	N/A	↑LRRK2 protein and the phosphorylation at Ser935	Atashrazm et al., 2019
Human	Memory T cells (PD patients)	N/A	↓LRRK2 mRNA in CD4 ↑LRRK2 mRNA in CD8	Dhanwani et al., 2022

Yet, LRRK2 was involved in the immune regulation of PD in both peripheral systems and CNS (Lee et al., 2017). LRRK2 is most highly expressed in bone marrow cells such as monocytes, dendritic cells, and neutrophils, whereas minimally expressed in B cells and T cells (Gardet et al., 2010; Hakimi et al., 2011; Thevenet and Pescini Gobert, 2011; Daher et al., 2015; Cook et al., 2017). Using fluorescence-activated cell sorting (FACS) of white blood cell subtypes isolated from the venous blood of healthy people, the LRRK2 expression is found highest in neutrophils, followed by monocytes, and then B cells (Shutinoski et al., 2019). LRRK2 mRNA and protein levels in both macrophages and leukocytes *in vitro* were up-regulated after exposure to pathogens and inflammatory mediators such as IFN-γ, IFN-β, TNF-α, and IL-6 (Hakimi et al., 2011; Thevenet and Pescini Gobert, 2011; Kuss et al., 2014). Similarly, the levels of LRRK2 protein in PD patients’ B cells, T cells (CD4^+^, CD8^+^, and T regulatory cells), CD14^+^, and CD16^+^ monocytes were increased compared with healthy controls (Bliederhaeuser et al., 2016; Cook et al., 2017). Moreover, this study revealed that the expression of LRRK2 protein and its phosphorylated level at Ser935 were significantly increased in neutrophils of PD patients (Atashrazm et al., 2019). Very recently, Dhanwani et al. (2022) first discovered that memory T cells expressed LRRK2 in the peripheral blood of diagnosed motor PD, which was differentially expressed in CD4 and CD8 memory T cells. LRRK2 was downregulated in CD4 and upregulated in CD8 memory T cells in PD patients, indicating that the two kinds of T cells play opposing roles in PD-related T-cell autoimmunity.

## The Role of Leucine-Rich Repeat Kinase 2 in the Immunopathogenesis of Parkinson’s Disease

LRRK2 protein is a complex multi-domain protein with a size of 286 kDa and contains seven domains (Mills et al., 2018), which endows its sophisticated function during the immunopathogenesis of PD. For example, LRRK2 serves a variety of functions including protein translation, cytoskeleton remodeling, vesicle transport, autophagy, mitochondrial homeostasis, and so on. LRRK2-mediated neuroinflammation is tightly associated with those functions. It is well known that immune cells are indispensable for homeostasis, immune defense, and tissue repair, hence the migration of immune cells to the injury sites is a key factor of disease progression in PD. *LRRK2* mutants display significant impairments of selective forms of autophagy (i.e., mitophagy and chaperone-mediated autophagy) and lysosomal function (Albanese et al., 2022). As the energy factories of cells, mitochondria are involved in the regulation of cell growth, apoptosis and other processes, and mitochondrial dysfunction is related to a variety of neurodegenerative diseases. Here, we will summarize the versatile functions of LRRK2 in immune cells that may contribute to more comprehensive of PD therapy.

### Migration

Recent studies have shown that LRRK2 mediates the process of cell migration to sites of injury or infection. LRRK2 exerts a reducing effect on microglial migration. Microglia carrying *LRRK2*^*G*2019*S*^ showed ADP-induced motor retardation and delayed injury isolation, and *LRRK2*-knockdown microglia are highly motile compared with control cells (Choi et al., 2015; Figure 3). Moreover, *LRRK2*-null microglia migrated faster and traveled a longer distance toward the regulation of chemokine (C-X3-C) receptor 1 (CX3CR1)-mediated signaling pathways (Ma et al., 2016; Figure 3). It has been shown that microglia carrying the *LRRK2*^*G*2019*S*^ mutation show ADP-induced motor deficits. The underlying mechanism is that LRRK2 binds focal adhesion kinase (FAK) and phosphorylates its Thr-X-Arg/Lys (TXR/K) motif(s), resulting in a decrease in FAK’s pY397 phosphorylation (Choi et al., 2015; Figure 3).

Similarly, the phenotypic and functional characteristics for migration have been observed in immune cells. The migration ability of human monocytes differentiated from iPSCs carrying the G2019S mutation has a moderate defect compared with the control (Speidel et al., 2016). Conversely, LRRK2 deficiency in mouse dendritic cells mainly manifested the enhancement of migration by interfering with ORAI2 (Yan et al., 2019; Figure 3). In addition, RNA sequencing (RNA-seq) profiled transcriptomic changes of activated primary macrophages from both WT and *LRRK2*-knockout (KO) mice, and found that LRRK2 may inhibit cAMP/EPAC-1 activity, further restricting movement and effective migration of cells to the site of neuronal injury (Levy et al., 2020; Figure 3).

However, in another study, both *in vitro* and *in vivo* experiments on the myeloid cells expressing *LRRK2*^*G*2019*S*^ showed that *LRRK2* mutant enhanced chemotaxis through the association between LRRK2 and actin-regulatory protein, which is blocked by the treatment of LRRK2 kinase inhibitors (Moehle et al., 2015; Figure 3). In conclusion, LRRK2 affects the migration of microglia and other immune cells, but the functions of elevated LRRK2 expression or carrying *LRRK2* mutations in different cell types might have double-faceted roles.

### Autophagy

Autophagy refers to the recycling of intracellular components by degrading dysfunctional or damaged proteins and organelles within the cell. Multiple studies have shown that LRRK2 protein is related to impaired autophagy (Schapansky et al., 2014; Cherubini and Wade-Martins, 2018; di Domenico et al., 2019). LRRK2 was first reported to regulate autophagy specifically in immune cells where monocytic cell lines (BV2 and RAW264.7) increased LRRK2 translocation to autophagosome membrane after LPS stimulation, whereas loss of LRRK2 lead to autophagic deficits (Schapansky et al., 2014). Inhibition of LRRK2 kinase activity also reduced autophagic degradation, reminiscent of the importance of the kinase domain in regulating autophagy. In consistent with this observation, toll-like receptor 4 (TLR4)-mediated microglial activation showed increased expression of endogenous LRRK2 and further increased autophagic flux (Moehle et al., 2012; Schapansky et al., 2014). In *LRRK2*-KO macrophages, autophagy was defective and not able to be converted to glycolytic metabolism after LPS treatment, whereas the pathogenic *LRRK2*^*G*2019*S*^ mutant caused overwhelming autophagy (Nabar et al., 2021; Figure 3). Collectively, the above studies have demonstrated that LRRK2 is significantly beneficial for autophagy.

Conversely, some studies have gave rise to opposite results. Compared with the normal control, the iPSCs derived astrocytes from PD patients carrying the G2019S mutation showed a significant reduction in autophagic flux (di Domenico et al., 2019). PD-related *LRRK2* mutations (G2019S, R1441C, or Y1699C) in astrocytes disrupted the function of lysosomes in a kinase-dependent manner. The autophagy flux could be restored upon *LRRK2* knockdown or supplementation with LRRK2 kinase inhibitors (Henry et al., 2015). *LRRK2* overexpression caused inactivation of Beclin-1 and inhibition of autophagy in mouse bone-marrow-derived dendritic cells (Manzoni et al., 2016; Figure 3). Moreover, Chen et al. (2018b) found that the Mn exposure up-regulated microglial LRRK2 expression both *in vitro* and *in vivo*, accompanied by autophagy dysfunction, which could be reversed by the inhibition of LRRK2. Also, the reduction of autophagy marker, LC3-II, has been demonstrated in cultured bone marrow-derived macrophages (BMDM) from mice carrying *LRRK2*^*R*1441*C*^ mutation (Hakimi et al., 2011). In summary, the link between LRRK2 and autophagy has been widely studied, albeit with conflicting results. However, the specific mechanisms of how LRRK2 regulates immune autophagy and lysosomal degradation are yet completely understood, which requires more efforts and explorations.

### Phagocytosis

Enhanced phagocytosis is associated with increased kinase activity in both macrophages and microglia from PD patients and mice. LRRK2 regulates the phagocytic responses by binding and phosphorylating the Thr470 site of actin-cytoskeletal regulator, WASP-family verprolin-homologous protein-2 (WAVE2) (Kim et al., 2018; Figure 3). Studies have shown that the RAB protein network was essential for the maturation of phagosomes by proteomics analysis (Gutierrez, 2013). Specifically, RAB5A co-localizes with the complex, formed during the phagosome-early endosome fusion of LRRK2 and WAVE2 in BMDM (Kim et al., 2018). A recent *in vitro* study indicates that LRRK2 and WAVE2 are important mediators of cytokine production and cytoskeletal rearrangement necessary for microglia-induced neurotoxicity (Fenner et al., 2022). LRRK2 is also required for the recruitment of RAB8A and RAB10 to phagosomes in macrophages derived from hPSCs (Lee et al., 2020; Figure 3). Meanwhile, endogenous LRRK2 is involved in the process of phagocytosis and bacterial killing in human immune cells; *LRRK2* knockdown could dampen the production of ROS (Gardet et al., 2010). Multiple studies have also demonstrated that microglia from mice with *LRRK2*^*G*2019*S*^ mutant showed increased cell activity and phagocytic responses *in vitro* (Choi et al., 2015; Kim et al., 2018; Dwyer et al., 2020). After trans activator of transcription (Tat) treatment, LRRK2 kinase inhibitors can inhibit the expression of key receptors for phagocytosis (brain-specific angiogenesis inhibitor 1, BAI1), thereby preventing phagocytosis in murine microglial line BV2 (Marker et al., 2012).

On the contrary, reducing LRRK2 kinase activity in both mouse and human macrophages could control the replication of *Mycobacterium tuberculosis* by enhancing the maturation of phagosomes, regardless of autophagy (Hartlova et al., 2018). Knockout of *LRRK2* in microglia increased the number of RAB5-positive endosomes, which later increased levels of the uptake and clearance of α-synuclein aggregates (Maekawa et al., 2016). In summary, for different models and immune cells, dissimilar mutations of *LRRK2* play disparate regulatory roles in phagocytosis.

### Mitochondria

Mitochondrial dysfunction is one of the pathological manifestations of neurodegenerative diseases such as PD. LRRK2 in mainly located in the cytoplasm, while around 10% is present in the mitochondria of cells with *LRRK2* overexpression (Biskup et al., 2006). The precipitation, super-resolution structured illumination microscopy (SR-SIM), and 3D virtual reality (VR) assisted colocalization analysis showed that the overexpression of *LRRK2*^*G*2019*S*^ leads to the formation of large perinuclear aggregates colocalized with the TOM (the translocase of outer mitochondrial membrane) complex (Neethling et al., 2019; Figure 3). The research models of LRRK2 and mitochondria mainly focus on autopsy tissues from PD patients and animal models carrying *LRRK2* mutants, as well as various cell models of LRRK2-related diseases. So far, multiple manifestations of mitochondrial dysfunction in PD have been revealed, including increased oxidative stress, decreased mitochondrial membrane potential, decreased ATP production, aggravated mitochondrial DNA (mtDNA) damage, mitochondrial elongation, mitochondrial fragmentation, and damaged mitochondrial phagocytosis (Singh et al., 2019).

Pathogenic *LRRK2* mutants are associated with increased sensitivity to oxidative stress that lead to extensive cell death. The mutations could also impair the antioxidant defense of mitochondria through several different mechanisms. In BV2 cells and primary microglia cells, *LRRK2*^*G*2019*S*^ revealed a decrease in mitochondrial area and shortage of microglial processes through DRP1 in a kinase-dependent manner, thereby initiating pro-inflammatory responses (Ho et al., 2018; Figure 3). The LRRK2 deficiency changed the expression of natural immune genes in macrophages driven by mitochondrial stress, suggesting that LRRK2-dependent mitochondrial defects may be involved in the regulation of innate immunity (Weindel et al., 2020).

The *LRRK2*^*G*2019*S*^ mutation also causes mtDNA damage, which is dependent on LRRK2 kinase activity. Specifically, the mtDNA damage in immune cells derived from PD patients with the *LRRK2*^*G*2019*S*^ mutation was significantly observed, and the mitochondrial mass and mtDNA copy number were also increased (Gonzalez-Hunt et al., 2020; Figure 3). Several studies have shown that the transport and movement of axon mitochondria are inhibited in mutants carrying *LRRK2*^*R*1441*C*/*G*2019*S*^ (Godena et al., 2014). LRRK2 kinase inhibitors failed to alleviate those defects related to mitochondrial transport (Hsieh et al., 2016; Thomas et al., 2016; Schwab et al., 2017). A recent study confirmed that microglia transport mitochondria through tunnel nanotubes to co-degrade with neighboring microglia. However, the transfer strategy is compromised in the microglia carrying *LRRK2*^*G*2019*S*^ (Scheiblich et al., 2021).

Moreover, the extra roles of LRRK2 in mitochondrial biology has yet studied exclusively in immune cells. For example, recent studies have shown that LRRK2 affected the autophagy pathway of mitochondria by direct and indirect effects. The *LRRK2*^*G*2019*S*^ mutation played a direct role in delaying mitochondrial phagocytosis by disrupting the removal of MIRO (Hsieh et al., 2016). Conversely, LRRK2 caused mitochondrial autophagy defects by affecting mitochondrial membrane potential damage (Su et al., 2015) and susceptibility to mitochondrial toxins (Novello et al., 2021). LRRK2 directly participates in the calcium homeostasis of mitochondria. The loss, inhibition, and mutation of LRRK2 lead to impaired mitochondrial Ca^2+^ buffering capacity, which leads to damage and degradation of mitochondrial function (Cherra et al., 2013; Schwab and Ebert, 2015; Ludtmann et al., 2019). In addition, LRRK2 has also been shown to alter mitochondrial dynamics and quality control. Basically, the fission and fusion process of mitochondria is regulated by stringent molecular mechanisms of dynein-related GTPases and WD40 repetitive proteins. Interestingly, LRRK2 contains both GTPase and WD40 domains, which structurally hints the importance of LRRK2 in mitochondrial dynamics (Walter et al., 2019).

Mitochondria are the main sites of intracellular energy synthesis and play an important role in maintaining the normal physiological functions of cells. According to the above studies, LRRK2 has been shown to be involved in various mitochondrial pathways, however, there still exist several questions in studying the link between LRRK2 and mitochondrial dysfunctions specifically in immune cells including microglia. Furthermore, how mitochondrial dysfunction mediates the immunopathogenesis of PD remains to be fully elucidated.

## Immunometabolism

Metabolism is the last step in the research pipeline, explaining the association of discovered metabolites with biological processes or biological states. In a sense, it may be considered that metabolism better reflects the real situation of the interaction between genes and the environment. Notably, as an important branch of systems biology, metabolomics has many unique advantages compared with other genomics, such as transcriptomics, and proteomics. For example, metabolites can be directly correlated to phenotypic changes in an organism while being more easily detected and metabolites’ functions clearer. Immunometabolism is an emerging field to study the interaction between immune cells and metabolic processes (Artyomov and Van den Bossche, 2020). With the latest advances, several findings highlighted multiple shared pathways between immune and metabolic processes, which are highly unified yet interdependent.

Basically, in resting cells, the major metabolic pathway is oxidative phosphorylation (OXPHOS), in which glucose is metabolized to pyruvic acid by glycolysis, and most pyruvic acid enters the tricarboxylic acid (TCA) cycle (Paolicelli and Angiari, 2019). However, in highly proliferative or tumor cells, the metabolic pathway switches from OXPHOS to aerobic glycolysis, which is known as the Warburg effect (Warburg et al., 1927). Importantly, this metabolic reprogramming mechanism also recapitulates in innate immune cells, including microglia, activated after LPS stimulation (Kelly and O’Neill, 2015). Moreover, LRRK2 regulates the glycolytic switch and cytokine production in response to stimulation by IFN-γ in microglia (Panagiotakopoulou et al., 2020).

So far, metabolomics in immune cells is substantially more challenging than transcriptomics and proteomics. Studies have shown that astrocytes derived from patient-specific iPSCs with *LRRK2*^*G*2019*S*^ mutation have lower glycolysis levels than from healthy people, and the metabolic profile has occurred significant changes (Sonninen et al., 2020). To more truly and accurately reflect the role of LRRK2 in PD, studies have directly performed metabolite testing on patients with *LRRK2* mutations. The metabolomics analysis of the cerebrospinal fluid showed that six metabolic pathways changed: fatty acid metabolism, beta-oxidation of short-chain fatty acids (SCFAs), bile-acid metabolism, spermidine, and spermine biosynthesis, methionine metabolism, mitochondrial beta-oxidation of long chain fatty acids (LCFAs), and methionine metabolism. The study again proved that bile acid metabolism is one of the major abnormal metabolic pathways in PD patients carrying *LRRK2* mutations (Yilmaz et al., 2020). Other studies have tested the metabolomics of the plasma of patients with *LRRK2* G2019S or R1441G mutations. Compared with the control, they did not combine bile acids (cholic acid, lithocholic acid, and deoxycholic acid), and intermediate metabolites of purine bases (especially hypoxanthine) levels were found elevated (Yakhine-Diop et al., 2020). Moreover, a recent study also demonstrated that *LRRK2*-KO macrophages were unable to switch to glycolytic metabolism after LPS treatment (Nabar et al., 2021).

## Perspectives

**FIGURE 4:**
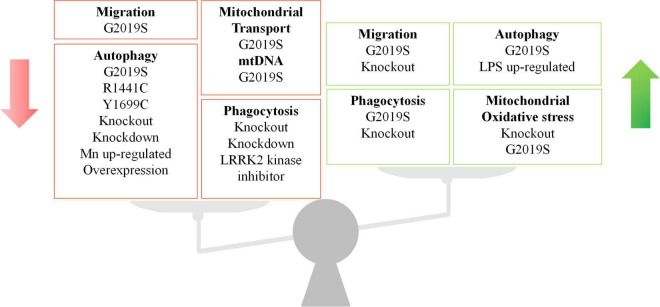
The double-faceted role of LRRK2 in different immune cell types and LRRK2 models. LRRK2 has been shown to regulate migration, autophagy, phagocytosis, and mitochondrial function in immune cells. The regulation role of LRRK2 expression is inconsistent across cell types and models. The green arrow indicates an increase and the red arrow indicates a decrease.

As an essential pathogenic gene, LRRK2 plays versatile roles throughout development of PD. However, LRRK2 seems to have diverse effects in different immune cells and PD models (Figure 4), which should be explored in future studies. For a long time, neuroscientists have largely focused on the role of LRRK2 in neurons, where the endogenous LRRK2 expression is low (Schapansky et al., 2014). As such, numerous studies have relied on the overexpression of LRRK2 in cell lines or animal models, which might not recapitulate the genuine physiological interaction of LRRK2 (Takagawa et al., 2018; Liu et al., 2020). Furthermore, there remain several unanswered questions about LRRK2, due to the complex structure of LRRK2 protein and the diversity of models in LRRK2-related research. As is well known, the phosphorylation level of LRRK2 is one of the conditions that researchers are more concerned about, especially the two phosphorylation sites of G2019S and R1441C. However, whether LRRK2 phosphorylation is beneficial or harmful in humans is still controversial. Since most of the data of LRRK2 phosphorylation were derived from the condition of overexpressed LRRK2, the studies on the phosphorylation of endogenous LRRK2, at a lower level, probably with more appropriate models, are required.

It is also highly desirable to identify novel signaling molecules/pathways that might regulate LRRK2 functions to provide theoretical benefits for PD treatment. Targeting LRRK2 pathways/axis is beneficial not only for patients with *LRRK2* mutations, but also idiopathic patients. As increased LRRK2 kinase activity is associated with both familial and sporadic PD patients, a large number of small molecules that can specifically inhibit kinase activity have been developed and launched in various clinical trials. At present, the search for therapeutically effective LRRK2 kinase inhibitors has made relatively optimistic progress, with two molecules in clinical trials and multiple alternatives in the pipeline (Azeggagh and Berwick, 2022). Interestingly, LRRK2 expression in peripheral blood mononuclear cells may be related to type II interferon response, and is also induced by interferon in T cells. Thus, targeting LRRK2 may not only affect the nervous system, but also be involved in complex autoimmune processes (Dhanwani et al., 2022).

## Author Contributions

YT conceived and designed the study. MZ and YT prepared the draft and figures. All authors have read, revised, and agreed to the published version of the manuscript.

## Conflict of Interest

The authors declare that the research was conducted in the absence of any commercial or financial relationships that could be construed as a potential conflict of interest.

## Publisher’s Note

All claims expressed in this article are solely those of the authors and do not necessarily represent those of their affiliated organizations, or those of the publisher, the editors and the reviewers. Any product that may be evaluated in this article, or claim that may be made by its manufacturer, is not guaranteed or endorsed by the publisher.
